# Phylogenetic Diversity and Environment-Specific Distributions of Glycosyl Hydrolase Family 10 Xylanases in Geographically Distant Soils

**DOI:** 10.1371/journal.pone.0043480

**Published:** 2012-08-17

**Authors:** Guozeng Wang, Kun Meng, Huiying Luo, Yaru Wang, Huoqing Huang, Pengjun Shi, Peilong Yang, Zhifang Zhang, Bin Yao

**Affiliations:** 1 Key Laboratory for Feed Biotechnology of the Ministry of Agriculture, Feed Research Institute, Chinese Academy of Agricultural Sciences, Beijing, People's Republic of China; 2 Biotechnology Research Institute, Chinese Academy of Agricultural Sciences, Beijing, People's Republic of China; Indiana University, United States of America

## Abstract

**Background:**

Xylan is one of the most abundant biopolymers on Earth. Its degradation is mediated primarily by microbial xylanase in nature. To explore the diversity and distribution patterns of xylanase genes in soils, samples of five soil types with different physicochemical characters were analyzed.

**Methodology/Principal Findings:**

Partial xylanase genes of glycoside hydrolase (GH) family 10 were recovered following direct DNA extraction from soil, PCR amplification and cloning. Combined with our previous study, a total of 1084 gene fragments were obtained, representing 366 OTUs. More than half of the OTUs were novel (identities of <65% with known xylanases) and had no close relatives based on phylogenetic analyses. Xylanase genes from all the soil environments were mainly distributed in Bacteroidetes, Proteobacteria, Acidobacteria, Firmicutes, Actinobacteria, Dictyoglomi and some fungi. Although identical sequences were found in several sites, habitat-specific patterns appeared to be important, and geochemical factors such as pH and oxygen content significantly influenced the compositions of xylan-degrading microbial communities.

**Conclusion/Significance:**

These results provide insight into the GH 10 xylanases in various soil environments and reveal that xylan-degrading microbial communities are environment specific with diverse and abundant populations.

## Introduction

Xylan is the second most abundant polysaccharide in nature, accounting for approximately one-third of all renewable organic carbon on earth [1]–[3]. It is made up of homopolymeric backbone chain of β-1,4-linked xylopyranose units with substitution of side chains at different positions [4]. Complete hydrolysis of xylan requires a large variety of cooperatively acting enzymes [3], [5]; among them, endo-1,4-β-d-xylanase (EC 3.2.1.8) is a crucial component that cleaves the backbone of xylan. Xylanases belonging to glycosyl hydrolase (GH) families 10 and 11 are the most abundant and have been studied extensively, and enzymes with xylanase activity have also been found in GH 5, 7, 8 and 43 (http://www.cazy.org/fam/acc_GH.html) [3], [6] and GH 30 [7], [8]. GH 10 and 11 xylanases are truly distinct; they share no similarity at the amino acid level and have different three-dimensional structures [9] and mechanisms of action [10]. In the Pfam database (http://pfam.sanger.ac.uk/), more GH 10 xylanases (1093 sequences) are identified in more microbial sources than GH 11 xylanases (527 sequences). In addition, most of the xylanase genes from rumen microbiome [11], termite hindgut microbiome [12] and tundra soil [13] belong to GH 10. Therefore we specified on GH 10 xylanase for further studies.

Soil harbors abundant and diverse microorganisms which are critical to maintain soil functions such as soil structure formation, decomposition of organic matter and cycling of carbon, nitrogen, phosphorus, and sulphur [14], [15]. Plant-litter input constitutes the main source of energy and matter for the extraordinarily diverse community of soil microorganisms connected by highly complex interactions in terrestrial ecosystem [16], [17]. Xylan as one major structural polysaccharide in plant cells, its degradation is a key step in carbon cycle in the soil environment.

Soil microorganisms represent rich resources of novel natural products like antibiotics and biocatalysts [18]. Compared with the traditional isolation and pure cultivation approach that only reflects limited information of soil microbial communities, culture-independent molecular methods are more powerful to explore the functional genes of the uncultured majority in the soil [19]. Two strategies are usually used to obtain xylanase genes from soil genomic DNA. One is metagenomic library construction and screening, e.g. Hu *et al*. [20] screened several novel xylanase genes from the soil metagenomic library. The other is PCR based molecular methods that are used to obtain xylanase gene fragments and full-length genes by using inverse PCR or genome walking. Sunna and Bergquist [21] cloned a novel extremely thermostable xylanase from a hot pool environmental DNA sample. Both strategies have its advantages and disadvantages [22]. Our previous studies sought to gain an insight into the diversity of GH 10 and 11 xylanases in alpine tundra soil and rumen using degenerate PCR, gene cloning from environmental DNA and functional verification [13], [23]. By using the same methods, the work presented in this report further addresses two main research goals: (i) to explore the similarities and differences of GH 10 xylanases genes from more soil samples with distinct environmental characteristics; (ii) to identity the factors that influence the diversity and distribution of xylanases in nature.

## Results and Discussion

### Physicochemical characters of five soil samples

Five vegetated and fell-field soil habitats spanning 22° of latitude and 34° of longitude were sampled to investigate the diversity of GH 10 xylanase genes of different terrestrial habitats. They were two soils (SS and GS) from Tianshan Mountain that represented low temperature soil environment with different vegetation types, a common agricultural soil from a maize field (FS), a hot spring sediment (HS) to represent high temperature soil environment, and a pond sediment (PS) and a muddy mangrove soil (MS) that represented freshwater and seawater environments, respectively. Details of their locations and physicochemical properties are given in Table 1.

**Table 1:** Physicochemical characters of six soils and GH 10 xylanase fragment sequences obtained.

Soil	Location	Habitat	Temperature (°C)	pH	Total organic carbon (mg/g)	Total nitrogen (mg/g)	C/N ratio	Clones sequenced	Sequences recovered	OTUs
MS	21° 29′ N, 109° 45′ E	Mangrove swamp	19	5.52	33.3		2.2	231	190	48
HS	23° 26′ N, 103° 08′ E	Hot spring	74	8.27	1.2	0.07	17.5	218	156	34
PS	30° 42′ N, 120° 55′ E	Pond	15	7.36	9.7	1.3	7.6	246	210	64
FS	27° 55′ N, 116° 22′ E	Farmland	6	7.12	13.2	1.5	8.8	227	188	61
SS	43° 06′ N, 86° 50′ E	Snow lotus	2	8.22	56.5	4.1	13.8	235	193	63
GS	43° 06′ N, 86° 50′ E	Glacier	2	6.89	33.8	2.6	12.9	216	147	96
Total								1373	1084	366

### Sequence analysis of GH 10 xylanase gene fragments

By using the total soil genomic DNA of each environmental sample as the template and the CODEHOP primers X_10_-F and X_10_-R specific for GH 10 xylanases [13], PCR products of the expected size, about 260 bp, were amplified and used to construct five clone libraries. After random confirmation of 300 clones from each library by PCR with primers M13F and M13R, a total of 1157 clones were sequenced; of them, 937 sequences were identified to be GH 10 xylanase gene fragments based on BLASTx analysis and contained the conserved residue Asn of GH 10 xylanases [24]. After removing the redundant fragments of each soil environment with Cd-hit program [25], 270 sequences were defined as distinct fragments with less than 95% identity (see Table S1, S2, S3, S4, S5).

**Figure 1:**
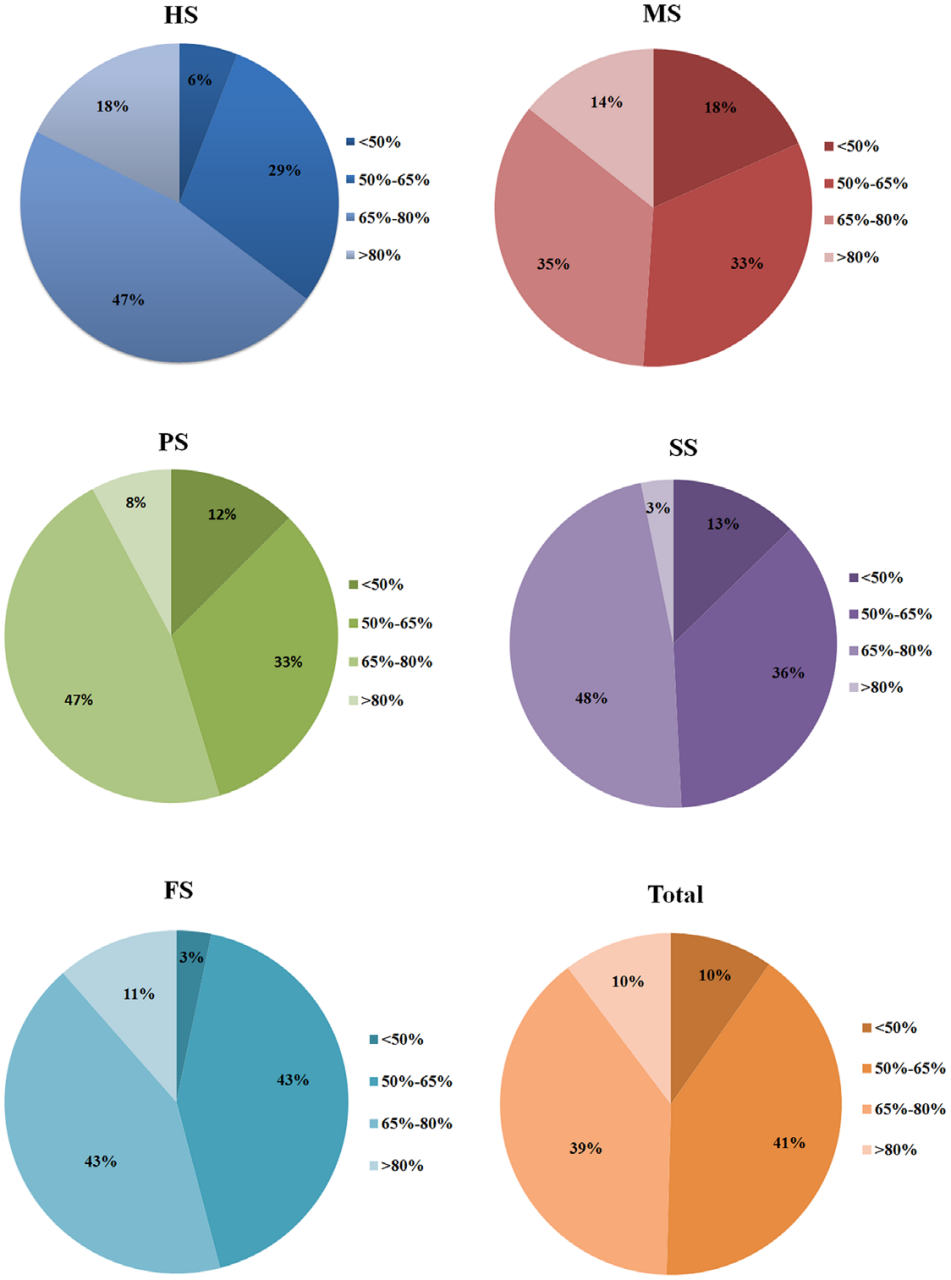
Amino acid sequence identities of GH 10 xylanase gene fragments from six soil environments to the known xylanases. Each sequence was analyzed with NCBI BLASTp (version 2.2.13) against the GenBank nr database. An E-score (expect value) cutoff of 10^–10^ (default) was applied and the top BLASTp hit to the known xylanases was collected. PS: pond sediment; MS: mangrove soil; HS: hot spring soil; FS: farmland soil; SS: snow lotus soil; GS: glacier soil.

Our previous study had identified 96 distinct GH 10 xylanase fragments in the tundra soil (GS) [13] that had different vegetation from SS. To compare the effects of vegetation on xylanase diversity and distribution, we combined the GS data with other five soils' to give a total of 366 distinct GH 10 xylanase fragments. Based on the BLASTp analysis, most sequences had low identities (< 65%) with known xylanases in GenBank (Figure 1), implying that these xylanases may be as yet undiscovered. Xylanase sequences from different soil environments varied in sequence identity distributions. About 18% of the distinct sequences from soil HS had the identity of more than 80% with known GH 10 sequences while only 3% in soil SS. Soil MS harbored the most novel sequences (identities <50%), representing 19% of the distinct sequences, and FS had the least, only 3% of the distinct sequences. Moreover, insertion or deletion of amino acids frequently occurred in this region. The lengths of GH 10 gene fragments displayed a wide range of variation (from 80 to 106 amino acids), and 57% of the distinct sequences had a length of 84–86 amino acids (Figure S1). The shortest sequence was PS33 (80 amino acids) that had 42% protein identity with a GH 10 protein from *Clavibacter michiganensis* [26]. The longest one was PS18 that contained 106 amino acids and had a maximum protein identity of 70% with *Prevotella ruminicola* xylanase [27]. These results suggested that our culture-independent molecular methods are efficient and reliable to retrieve xylanase genes of various lengths from different soil environments.

### High genetic diversity of GH 10 xylanases in the soils

**Figure 2:**
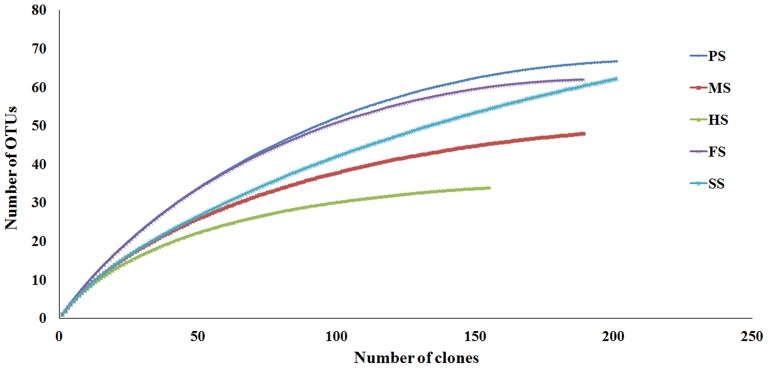
Rarefaction curves of GH10 xylanase fragments from six soil environments. The gene types were defined at the 6% cut-off. The curves represent the number of gene types as a function of total clones sequenced in libraries constructed from six soil genomic DNAs.

Using Distance-based OTU and Richness determination (DOTUR) software [28], rarefaction curves for GH 10 xylanases in each sample were calculated based on 6% cut-offs (Figure 2). The flat rarefaction curves in Figure 2 suggested that we have sequenced enough clones to account for the overall genes. Based on the UPGMA (average neighbor) clustering algorithm implemented in DOTUR, a total of 270 OTUs were identified (Table 1), 63 in soil SS, 34 in soil HS, 48 in soil MS, 64 in soil PS and 61 in soil FS. It meant that more GH 10 xylanase genes were present in the soil PS and much less in the soil HS. The most abundant OTU in each soil environment was different. HS3, the most abundant OTU in soil HS, represented about 20% (31/156) of all the sequences (Table S1) and had the highest identity (94%) with the xylanase sequence from the extreme thermophile *Dictyoglomus thermophilum* Rt46B.1 [29]. The most abundant OTUs in soil MS and PS were MS16 (17/190; Table S2) and PS159 (30/210; Table S3), respectively, both of which had the highest identities with the xylanase of *Prevotella bergensis* DSM 17361. The most abundant OTUs in soil SS and FS were SS153 (17/193; Table S4) and FS97 (13/188; Table S5), respectively; they had the highest identities with the xylanases from *Verrucomicrobiae bacterium* DG1235 and *Ktedonobacter racemifer* DSM 44963, respectively. The distinction of the most abundant OTUs in each soil implied that their major xylan-degrading microorganisms are different from each other. Moreover, the most abundant OTUs of each soil were found to be environment specific. For example, the most dominant OTU in the HS was closely related to the xylanase of the extreme thermophile *D. thermophilum* Rt46B.1 [29]. The most dominant OTUs in the PS and MS were related to the xylanase from anaerobic *P. bergensis*, a bacterium usually found in gastroenteric environments [30]. In the SS, the most dominant OTU was found to be related to the xylanase from Verrucomicrobiae bacterium, which belongs to the phylum Verrucomicrobia that has been reported to be dominant in cold environments [31].

**Figure 3:**
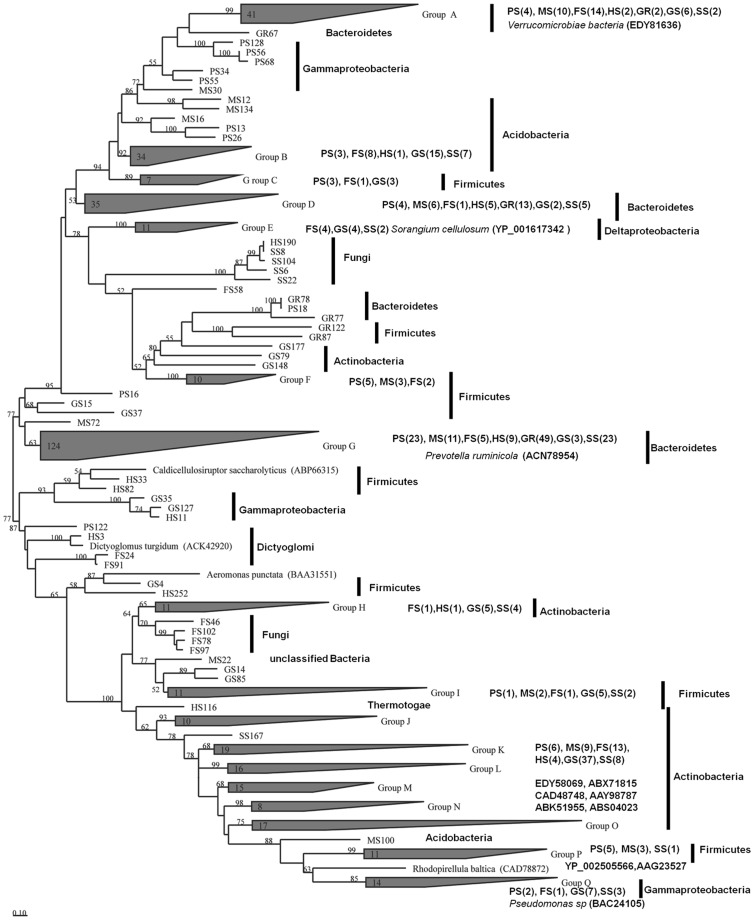
Phylogenetic tree constructed using ARB software using neighbor-joining method. A total of 19 representative sequences were randomly selected and used as references for phylogenetic tree construction. The lengths of the branches indicate the relative divergence among the amino acid sequences. Sequences with the same source were clustered together and the numbers of sequences in each soil were listed. The numbers at the nodes indicate bootstrap values based on 1000 replications and bootstrap values (>50) are displayed. The scale bar represents 0.1 amino acid substitution per position.

To understand the environmental impacts on xylanase diversity and distribution, goat rumen that represents a strict anaerobic environment together with the tundra soil [13], [23] were included for comparison analysis. A total of 418 sequences from seven samples and 16 representative reference sequences were used to construct phylogenetic tree (Figure 3) using ARB software [32]. Seventeen groups (A–Q) were separated based on the high bootstrap values; of them, only six groups A, E, G, K, L and O had reference sequences. Group G was the largest group containing 123 sequences from all seven environments along with the reference sequence of *P. ruminicola* [33]. Combining the sequences in group D, 38.8% of the distinct sequences (162/418) were closely related with GH 10 sequences of Bacteroidetes. Actinobacteria represented another main environmental xylanase producer, accounting for 22.0% of the sequences (92/418; group H, J, K, L, M, N and O). Firmicutes and Proteobacteria were the third and fourth xylanase producers, respectively. Xylanase genes have been found in other microbial sources, such as Verrucomicrobiae, Acidobacteria, Dictyoglomi, Thermotogae, fungi and unclassified bacteria in different soil environments. The phylogenetic diversity of xylanase genes in this study might not be very exact because horizontal gene transfer between family 10 xylanases is a common phenomenon in environments [34], [35].

Our study suggested that GH 10 xylanase genes are mainly distributed in bacteria in soil environments. Based on the collection of GH 10 sequences in Pfam database, Firmicutes are the main microbial source of xylanase production, accounting for 23% of the sequences, and Bacteroidetes constitute only 11%. These data are significantly different from our results. The reason might be that most of the sequences collected by the Pfam database are from cultured microorganisms while uncultured microorganisms are the main xylanase producer in soil environments. Moreover, when we compared the soil xylanase genes with those from rumen microbiome [11] and termite hindgut microbiome [12], we found that xylanases from soils are more diverse. GH 10 xylanase genes from the soil environments under study were widely distributed in the phyla of Bacteroidetes, Proteobacteria, Acidobacteria, Firmicutes, Actinobacteria, Dictyoglomi and the fungi of Ascomycota, while GH 10 xylanase genes from rumen and termite hindgut were mainly distributed in the phyla of Bacteroidetes and Firmicutes, respectively. The results suggested that soil environment harbors a more complex microflora than gastrointestinal tract environments.

Bacteria and fungi are the key agents of xylan degradation, in which fungi have been supposed to play an important role in the initiation of plant fiber degradation during fermentative digestion [1], [2], [36]. However, fewer fungal GH 10 xylanase gene fragments were identified in this study. Only eight xylanase fragments from snow lotus soil, hot spring sediment and pond sediment were found to be related to the xylanases from Ascomycota, a phylum dominant in soils and important in xylan degradation [36]. This phenomenon has also been found in other environments, such as no fungal GH 10 xylanases in the rumen microbiome [11] and termite hindgut microbiome [12]. However, our unpublished data showed that more GH 11 xylanase fragment sequences (78/122) from the same soil environments were related to fungal instead of bacterial xylanases. According to the Pfam database (http://pfam.sanger.ac.uk/), more GH 11 sequences are from fungi (296/527), and more GH 10 sequences are from bacteria (870/1093). These facts suggest that bacteria and fungi are the main producers of GH 10 and 11 xylanases, respectively. Considering that GH 10 and 11 xylanases are distinct at structures and action modes [10], they must work in xylan degradation in a compatible instead of competitive way.

Three sequences from mangrove soil and farmland soil were closely related to the xylanase of extremely halophilic archaeon *Halorhabdus utahensis*, suggesting the existence of xylanase-producing Archaea in these soil environments.

### Effects of environmental factors on GH 10 xylanase in the soils

**Figure 4:**
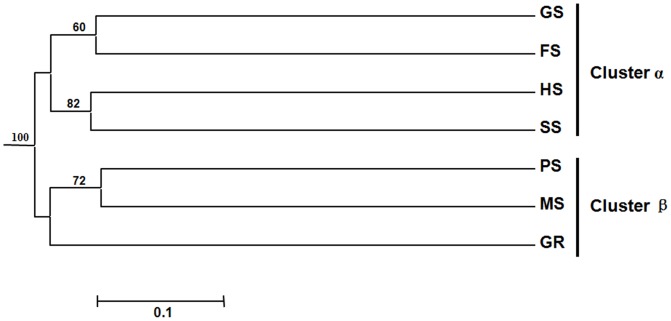
Cluster analysis of six soil samples with unweighted UniFrac algorithm.

The diversity, abundance and distribution of GH 10 xylanase genes in soils varied a lot from each other. The GH 10 xylanase genes in different soil samples were compared by UniFrac analysis [37] (Figure 4). High Jackknife Count (100) separated seven soil environments into two big clusters: cluster α containing soil GS, FS, HS and SS, and cluster β of other samples (PS, MS and GR). Soil SS and soil HS were clustered together although their soil characters differed a lot except for soil pH (Table 1). The GH 10 xylanase genes in soil GS were significantly different from soil SS although they were sampled from the same area but with different vegetation. The GH 10 xylanase gene fragments from soil PS, MS and GR were grouped together in cluster β. These three environments are all anaerobic. Studies have shown that environmental factors like pH, oxygen content, temperature, organic matter and vegetation might have great effect on the distribution and diversity of functional genes [38]–[40]. In this study, we found that pH and oxygen content are the most important geochemical factors influencing the xylanase diversity and distribution in soils. Microbial xylanases are often secreted as extracellular enzymes or are associated with the outer membrane [2], [3]. The extracellular location makes xylanases adapt to physicochemical conditions of the surrounding environment for efficient function.

Although the environmental factors differed a lot, 16 identical sequences were found between two or more soil samples (Figure S2). The same phenomenon has been reported by LeCleir *et* *al*. [41] in the study of chitinase genes in various aquatic habitats. For example, a fragment showing the highest similarity with a bifunctional GH 10 xylanase/CE 1 ferulic acid esterase of *P. ruminicola* [30] was found in six samples (MS150, GS122, HS7, SS121, FS144 and PS111); fragments MS36 and PS158, MS128 and PS36, MS13 and PS96, HS187 and SS32, and HS190 and SS8 were found to be identical. Another two pairs of sequences (HS262 and PS88, HS108 and PS21) were found both in soil HS and PS. Moreover, eight xylanase sequences from goat rumen were identical with that from soil environments (data not show). These results suggested that these xylanase genes are well conserved and broadly distributed in environment.

In conclusion, we identified large numbers of GH 10 xylanase fragments in five soil environments using culture-independent molecular methods. Most of the sequences have low identities with known xylanases and have no close relatives, suggesting the phylogenetic diversity and novelty of these xylanase genes. Compared to the xylanases from cultured bacteria, xylanases directly retrieved from soil environments have some specific distributions. The various diversity and distribution patterns of this functional gene in different soil environments suggest that xylan-degrading bacterial community is environment specific. Environmental factors such as pH and oxygen content may be the most significant geochemical factors that affected the distribution and diversity of microbial xylanase genes. Although we identified so many xylanase genes in soil environments, how and when these xylanase genes are expressed *in situ* is unknown. New approaches such as metatranscriptome analysis [42]–[44] will be powerful to solve these questions.

## Materials and Methods

### Ethics statement

No specific permits were required for the described field studies. All the soil samples were collected from the location that is not privately-owned or protected in any way. The field studies did not involve endangered or protected species because this study only concentrated on the soil samples.

### Collection of soil samples

Five soil types with different physicochemical characteristics were sampled in China (Table 1). Rhizosphere soil of snow lotus was sampled from tundra soil of Tianshan Mountain at an elevation of 3525 m. Hot spring sediment with temperature of 74°C was sampled from Jinping, Yunnan. Pond sediment was sampled from the pond which was used to fish culturing in Zhejiang. Field soil was sampled from the farmland in Jiangxi which had been used to grow maize. Mangrove soil was sampled from Guangxi. For all the soil samples, three soil cores (at a depth of 0–10 cm, 5 cm in diameter) were collected from each soil site and stored at –70°C before use. Soil parameters, including total organic carbon and total nitrogen, were determined by using Jackson's methods [45]. The pH of the soil sample was measured in a 1∶2 soil-water suspension.

### DNA extraction

Total soil genomic DNA was extracted using a modified sodium dodecyl sulphate (SDS)–based protocol specific for high molecular weight DNA from environmental samples [46]. Root material and small stones (if any) in the soil samples were removed by hand. One gram of each soil was added to a 2 ml screw-capped tube, followed by addition of 1 ml of lysis buffer (100 mM Tris–HCl, 100 mM EDTA, 1.5 M NaCl, 2% CTAB and 1% SDS) and complete mixing. Glass beads (0.2 ml of 0.1 mm in diameter and 0.1 ml of 0.5 mm in diameter) were added to the tubes, and agitated on MiniBead Beater-1 (Biospec, Bartlesville, OK) at the speed of 2500 rpm, 20 sec for three times. Then all the contents were removed to a 5 ml tube, another 3 ml of lysis buffer was added, mixed completely and incubated at 70°C for 2 h with gentle shaking every 20 min. After centrifugation at 16,000 ×*g* and 4°C for 10 min, genomic DNA was recovered by precipitation with 0.7 volume of isopropanol and dissolved in TE buffer. RNase was added to the crude soil DNA to digest RNAs (37°C, 1 h). The presence and size of the soil DNAs were determined by agarose gel electrophoresis with ethidium bromide staining. DNA samples were recovered, purified with the Omega Gel Extraction Kit (Norcross, GA) and stored at −70°C before use.

### PCR amplification, library construction and sequencing

The CODEHOP primers X_10_-F (5′-CTACGACTGGGAYGTNIBSAAYGA-3′) and X_10_-R (5′-GTGACTCTGGAWRCCIABNCCRT-3′) specific for GH 10 xylanase [13] were used to amplify GH 10 xylanase gene fragments by touchdown PCR from the purified metagenomic DNA of each soil sample. PCR products were visualized on an agarose gel, bands of the expected size (about 260 bp) were excised and purified using the Qiaquick gel extraction kit (Qiagen, Valencia, CA). Purified PCR products were ligated into vector pGEM-T Easy (Promega, Madison, WI), and then electroporated into *Escherichia coli* DH5α (TaKaRa, Tokyo, Japan) following the procedure recommended by the manufacturer. Three hundred positive transformants (white colonies) from each library were randomly picked for further confirmed by PCR with M13F (GTAAAACGACGGCCAGT) and M13R (GGATAACAATTTCACACAGGA) and sequenced by Sunbiotech (Beijing, China) using Sanger method with the ABI-3730 automatic sequencer (Life Technologies, Carlsbad, CA).

### Phylogenetic tree construction

Vector sequences introduced by automated Sanger sequencing machines were removed using Figaro software [47] (http://sourceforge.net/apps/mediawiki/amos/index.php?title=Figaro). Then the sequences were analyzed with NCBI BLASTx (version 2.2.13) against the GenBank nr database. An E-score (expect value) cutoff of 10^–10^ was applied. Nucleotide sequences identified as xylanase gene fragments were translated into amino acids by EMBOSS Transeq (http://www.ebi.ac.uk/emboss/transeq) and aligned with the known sequences in GenBank database at the protein level using ClustalW. Redundant amino acid sequences were removed using Cd-hit [25] with a 95% sequence identity cut-off.

ARB software [32] was used for construction of the sequence databases and phylogenetic trees (neighbor joining, Jukes-Cantor correction factor for protein with 1000 bootstrap replications). A total of 19 representative sequences were randomly selected and used as references for phylogenetic tree constructions. To test clustering, representative sequences and at least one environmental sequence from each group were used for phylogenetic analysis using minimum evolution (Jukes-Cantor correction) and maximum parsimony criteria with MEGA 4.0 [48].

### Rarefaction and Unifrac analysis

Rarefaction curves for GH 10 xylanases in each sample were calculated, respectively, using DOTUR software [28]. Distance matrices of the clones were calculated at the protein level using default parameters of protdist in PHYLIP (http://evolution.genetics.washington.edu/phylip.html). Sequences were then assigned to OTUs based on the UPGMA (average neighbor) clustering algorithm implemented in DOTUR with default parameters for precision (0.01) and bootstrap (1000), and rarefaction results at 6% protdist were calculated.

UniFrac was applied to examine the differences in the composition of GH 10 xylanase among samples [37]. First, phylogenetic tree was constructed for the xylanase gene sequences using ARB software as described above. The phylogenetic tree and the environment file were subsequently uploaded to UniFrac (http://bmf2.colorado.edu/unifrac/index.psp). In the present study, “P test significance" and “cluster environments" options were used.

### Nucleotide sequence accession numbers

The GH 10 xylanase gene fragments have been deposited into GenBank database under accession numbers FJ527914–FJ527975 for snow lotus soil, FJ918954–FJ919017 for pond sediment, FJ919018–FJ919065 for mangrove soil, FJ919066–FJ919094 for hot spring sediment, and FJ919095–FJ919155 for farmland soil.

## Supporting Information

Figure S1 **Length variation of GH 10 xylanase fragments from six soil environments.**
(TIF)

Figure S2 **Phylogenetic tree of identical xylanase fragments from two or more soil environments.** The phylogenetic tree was constructed using the neighbor-joining method (MEGA 4.0). The lengths of the branches indicate the relative divergence among the amino acid sequences. The numbers at the nodes indicate bootstrap values based on 1000 replications and bootstrap values (>50) are displayed. The scale bar represents 0.1 amino acid substitution per position.
(TIF)

Table S1 **The GH 10 xylanase gene fragments detected in the hot spring sediment (HS) and their closest relative based on amino acid sequence identity and similarity.**
(DOC)

Table S2 **The GH 10 xylanase gene fragments detected in the mangrove soil (MS) and their closest relative based on amino acid sequence identity and similarity.**
(DOC)

Table S3 **The GH 10 xylanase gene fragments detected in the pond sediment (PS) and their closest relative based on amino acid sequence identity and similarity.**
(DOC)

Table S4 **The GH 10 xylanase gene fragments detected in the snow lotus soil (SS) and their closest relative based on amino acid sequence identity and similarity.**
(DOC)

Table S5 **The GH 10 xylanase gene fragments detected in the farmland soil (FS) and their closest relative based on amino acid sequence identity and similarity.**
(DOC)
