# Supplementary figures and images for: Phylogenetic Diversity and Environment-Specific Distributions of Glycosyl Hydrolase Family 10 Xylanases in Geographically Distant Soils
Source: PLoS One. 2012 Aug 17;7(8):e43480. doi: 10.1371/journal.pone.0043480 (PMC3422244; doi:10.1371/journal.pone.0043480)

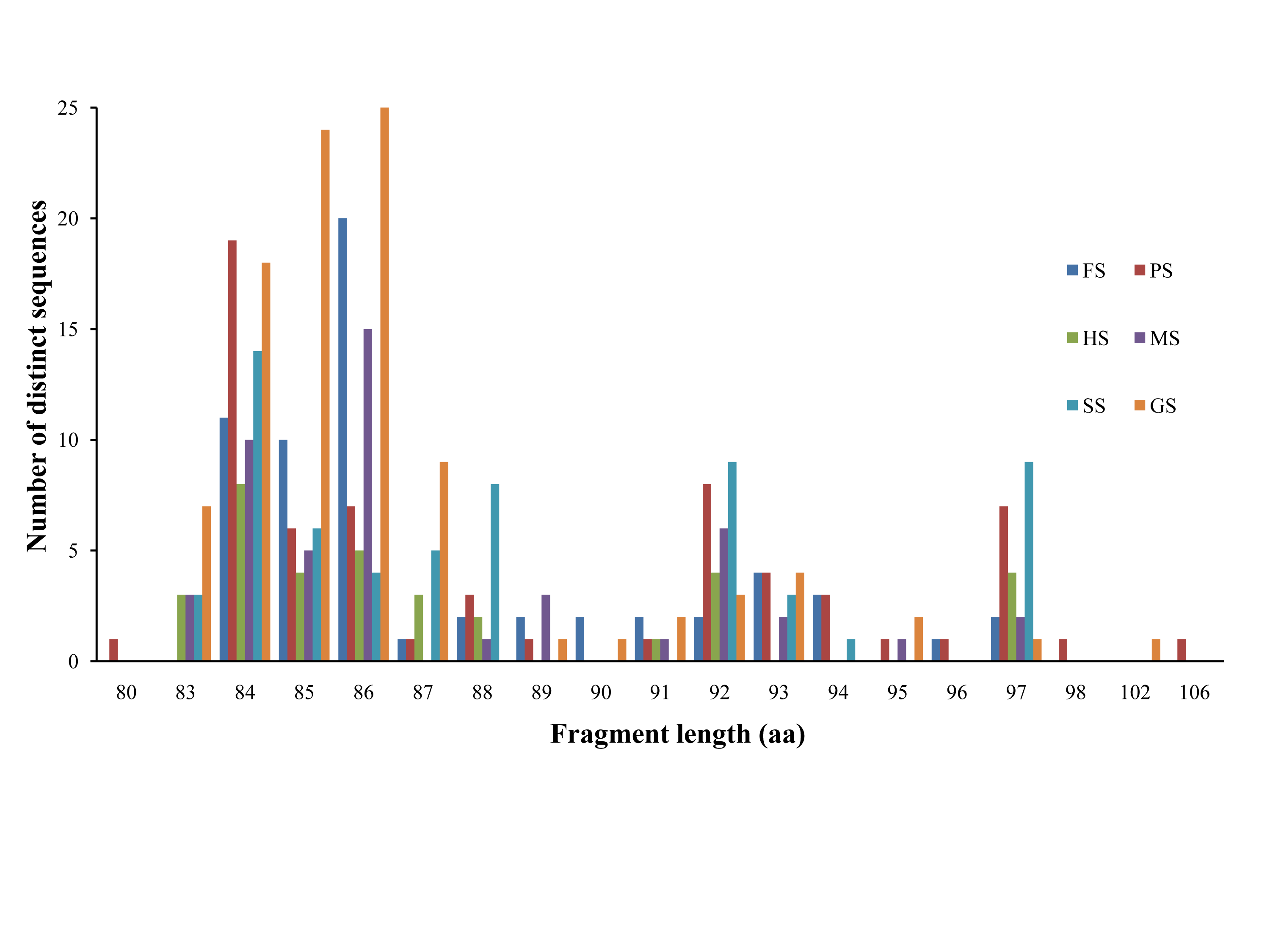

Supplement: Figure S1 — Length variation of GH 10 xylanase fragments from six soil environments. (TIF) [file pone.0043480.s001.tif]

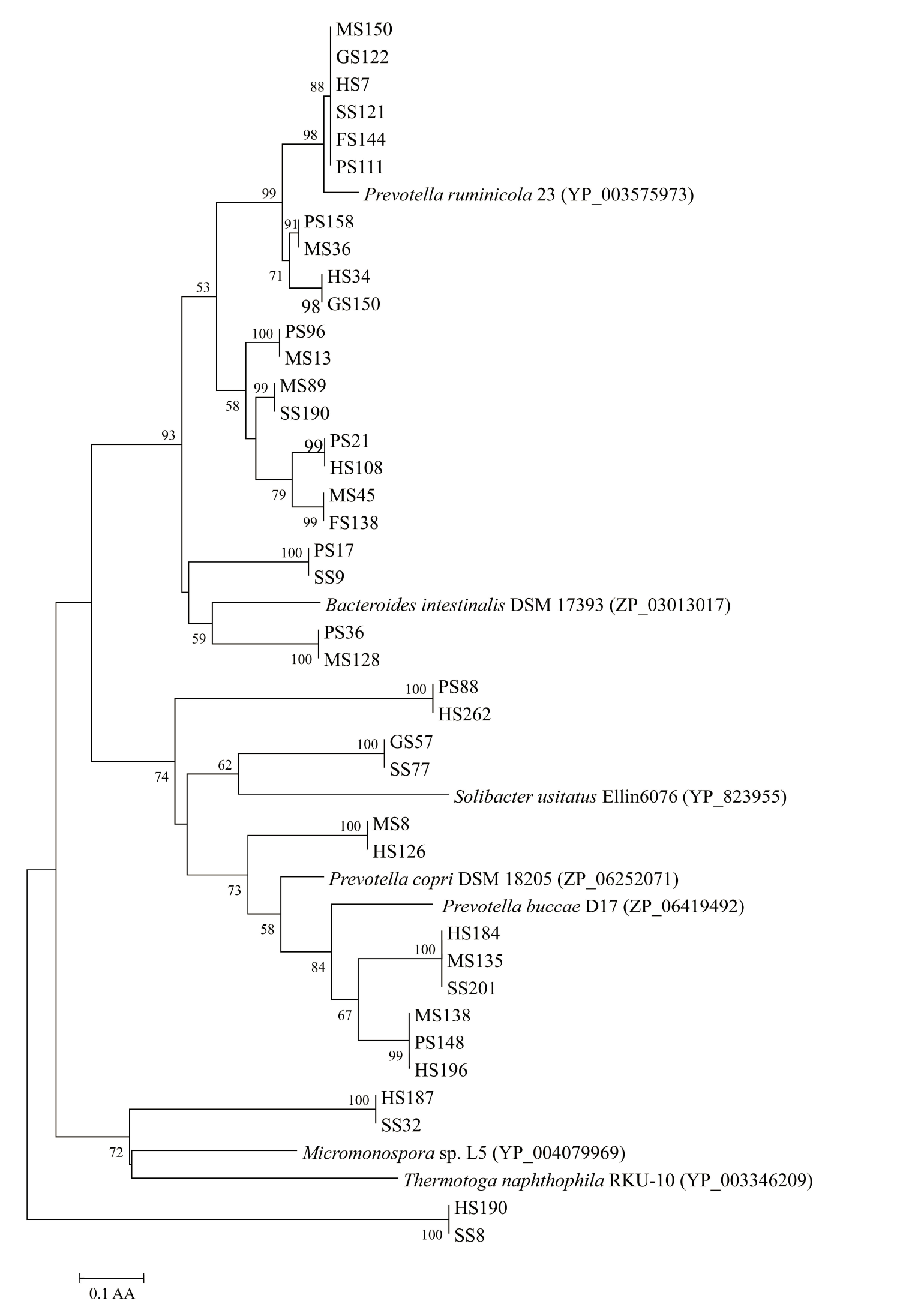

Supplement: Figure S2 — Phylogenetic tree of identical xylanase fragments from two or more soil environments. The phylogenetic tree was constructed using the neighbor-joining method (MEGA 4.0). The lengths of the branches indicate the relative divergence among the amino acid sequences. The numbers at the nodes indicate bootstrap values based on 1000 replications and bootstrap values (>50) are displayed. The scale bar represents 0.1 amino acid substitution per position. (TIF) [file pone.0043480.s002.tif]
