# Supplementary material for: Phylogenetic Diversity and Environment-Specific Distributions of Glycosyl Hydrolase Family 10 Xylanases in Geographically Distant Soils
Source: PLoS One. 2012 Aug 17;7(8):e43480. doi: 10.1371/journal.pone.0043480 (PMC3422244; doi:10.1371/journal.pone.0043480)
Supplement: Table S1 — The GH 10 xylanase gene fragments detected in the hot spring sediment (HS) and their closest relative based on amino acid sequence identity and similarity. (DOC) [file pone.0043480.s003.doc]

**Supplementary Table S1.** The GH 10 xylanase gene fragments detected in the hot spring sediment and their closest relatives based on amino acid sequence identity and similarity.

| OTU *^a^* | Protein size (amino acids) | Identity (%) | Amount of sequences | Closest relative (accession No.) |
| --- | --- | --- | --- | --- |
| HS190 | 97 | 37 | 3 | *Aspergillus fumigatus* Af293 (XP_754103) |
| HS55 | 92 | 63 | 2 | *Bacteroides cellulosilyticus* DSM 14838 (ZP_03676788) |
| HS93 | 88 | 75 | 4 | *B. cellulosilyticus* DSM 14838 (ZP_03678239) |
| HS87 | 91 | 36 | 7 | *Caldicellulosiruptor lactoaceticus* 6A (ZP_07736611) |
| HS82 | 86 | 94 | 2 | *Caldicellulosiruptor owensensis* OL (YP_004001877) |
| HS252 | 86 | 90 | 5 | *C. owensensis* OL (YP_004001532) |
| HS33 | 86 | 94 | 4 | *Caldicellulosiruptor saccharolyticus (*YP_001181178*)* |
| HS128 | 85 | 57 | 4 | *Clostridium papyrosolvens* DSM 2782 (ZP_05494069) |
| HS3 | 86 | 94 | 31 | *Dictyoglomus thermophilum* H-6-12 (YP_002251354) |
| HS74 | 86 | 55 | 4 | *Dictyoglomus turgidum* DSM 6724 (YP_002353534) |
| HS36 | 84 | 63 | 1 | *Leadbetterella byssophila* DSM 17132 (YP_003998360) |
| HS22 | 83 | 96 | 5 | *Meiothermus ruber* DSM 1279 (YP_003506085) |
| HS241 | 83 | 88 | 2 | *M. ruber* DSM 1279 (YP_003506085) |
| HS18 | 83 | 64 | 2 | *Micromonospora* sp. L5 (YP_004079969) |
| HS184 | 84 | 70 | 2 | *Prevotella buccae* ATCC 33574 (ZP_07881708) |
| HS126 | 84 | 72 | 2 | *Prevotella copri* DSM 18205 (ZP_06252071) |
| HS156 | 84 | 70 | 6 | *P. copri* DSM 18205 (ZP_06252071) |
| HS196 | 84 | 77 | 2 | *P. copri* DSM 18205 (ZP_06252071) |
| HS270 | 84 | 74 | 2 | *P. copri* DSM 18205 (ZP_06252071) |
| HS244 | 87 | 73 | 1 | *Paludibacter propionicigenes* WB4 (YP_004043427) |
| HS7 | 92 | 80 | 16 | *Prevotella ruminicola* 23 (YP_003575973) |
| HS34 | 92 | 71 | 14 | *P. ruminicola* 23 (YP_003575973) |
| HS108 | 97 | 66 | 2 | *P. ruminicola* 23 (YP_003575973) |
| HS120 | 92 | 77 | 2 | *P. ruminicola* 23 (YP_003575973) |
| HS172 | 88 | 71 | 2 | *P. ruminicola* 23 (YP_003575973) |
| HS202 | 97 | 66 | 3 | *P. ruminicola* 23 (YP_003575973) |
| HS243 | 97 | 67 | 3 | *P. ruminicola* 23 (YP_003575973) |
| HS9 | 84 | 59 | 3 | *Solibacter usitatus* Ellin6076 (YP_823955) |
| HS13 | 85 | 53 | 3 | *S. usitatus* Ellin6076 (YP_824087) |
| HS43 | 84 | 58 | 2 | *S. usitatus* Ellin6076 (YP_823955) |
| HS116 | 85 | 66 | 3 | *Spirochaeta thermophila* DSM 6192 (YP_003874722) |
| HS11 | 85 | 62 | 2 | *Teredinibacter turnerae* T7901 (YP_003072357) |
| HS187 | 87 | 56 | 6 | *Thermotoga naphthophila* RKU-10 (YP_003346209) |
| HS262 | 87 | 74 | 4 | *Verrucomicrobiae bacterium* DG1235 (ZP_05056496) |
| Total 34 |  |  | 156 |  |

*^a^* Sequence name was selected to represent each OTU.
