# Supplementary material for: Phylogenetic Diversity and Environment-Specific Distributions of Glycosyl Hydrolase Family 10 Xylanases in Geographically Distant Soils
Source: PLoS One. 2012 Aug 17;7(8):e43480. doi: 10.1371/journal.pone.0043480 (PMC3422244; doi:10.1371/journal.pone.0043480)
Supplement: Table S2 — The GH 10 xylanase gene fragments detected in the mangrove soil (MS) and their closest relative based on amino acid sequence identity and similarity. (DOC) [file pone.0043480.s004.doc]

**Supplementary Table S2.** The GH 10 xylanase gene fragments detected in the mangrove soil and their closest relatives based on amino acid sequence identity and similarity.

| OTU *^a^* | Protein size (amino acids) | Identity (%) | Amount of sequences | Closest relative (accession No.) |
| --- | --- | --- | --- | --- |
| MS13 | 93 | 67 | 3 | *Bacteroides cellulosilyticus* DSM 14838 (ZP_03676788) |
| MS25 | 95 | 63 | 3 | *B. cellulosilyticus* DSM 14838 (ZP_03676788) |
| MS118 | 92 | 64 | 8 | *B. cellulosilyticus* DSM 14838 (ZP_03678239) |
| MS128 | 86 | 65 | 10 | *B. cellulosilyticus* DSM 14838 (ZP_03678239) |
| MS89 | 93 | 66 | 2 | *Bacteroides eggerthii* DSM 20697 (ZP_03459580) |
| MS14 | 88 | 73 | 7 | *Bacteroides intestinalis* DSM 17393 (ZP_03013017) |
| MS109 | 89 | 44 | 3 | *Caldicellulosiruptor kronotskyensis* 2002 (YP_004025163) |
| MS41 | 86 | 46 | 5 | *Clavibacter michiganensis* (YP_001220829) |
| MS60 | 84 | 41 | 5 | *Clostridium cellulolyticum* H10 (YP_002504521) |
| MS43 | 84 | 45 | 1 | *Clostridium papyrosolvens* DSM 2782 (ZP_05496079) |
| MS49 | 89 | 42 | 3 | *Coprinopsis cinerea* okayama7#130 (XP_001829958) |
| MS30 | 84 | 63 | 3 | *Glaciecola mesophila* (ACN76857) |
| MS50 | 85 | 62 | 4 | *Halorhabdus utahensis* DSM 12940 (YP_003130024) |
| MS139 | 85 | 60 | 7 | *H. utahensis* DSM 12940 (YP_003130024) |
| MS98 | 89 | 44 | 6 | *Nocardiopsis dassonvillei* (YP_003680373) |
| MS120 | 86 | 50 | 1 | *N. dassonvillei* (YP_003680146) |
| MS16 | 84 | 66 | 17 | *Prevotella bergensis* DSM 17361 (ZP_06006687) |
| MS23 | 84 | 68 | 4 | *P. bergensis* DSM 17361 (ZP_06006687) |
| MS108 | 84 | 70 | 2 | *P. bergensis* DSM 17361 (ZP_06006687) |
| MS134 | 92 | 59 | 1 | *P. bergensis* DSM 17361 (ZP_06006687) |
| MS140 | 83 | 61 | 7 | *P. bergensis* DSM 17361 (ZP_06006687) |
| MS135 | 84 | 76 | 3 | *Prevotella buccae* D17 (ZP_06419492) |
| MS8 | 84 | 74 | 3 | *Prevotella copri* DSM 18205 (ZP_06252071) |
| MS138 | 84 | 76 | 2 | *P. copri* DSM 18205 (ZP_06252071) |
| MS4 | 97 | 63 | 2 | *Prevotella ruminicola* 23 (YP_003575973) |
| MS36 | 92 | 73 | 9 | *P. ruminicola* 23 (YP_003575973) |
| MS45 | 97 | 67 | 4 | *P. ruminicola* 23 (YP_003575973) |
| MS96 | 92 | 71 | 3 | *P. ruminicola* 23 (YP_003575973) |
| MS150 | 92 | 80 | 3 | *P. ruminicola* 23 (YP_003575973) |
| MS72 | 86 | 68 | 1 | *Paenibacillus* sp. HPL-001 (ACJ06666) |
| MS2 | 83 | 49 | 6 | *Solibacter usitatus* Ellin6076 (YP_824087) |
| MS100 | 86 | 55 | 3 | *S. usitatus* Ellin6076 (YP_825640) |
| MS88 | 91 | 49 | 1 | *Sorangium cellulosum '*So ce 56' (YP_001617342) |
| MS33 | 85 | 57 | 3 | *Streptomyces pristinaespiralis* ATCC 25486 (ZP_06913485) |
| MS38 | 86 | 80 | 2 | *Teredinibacter turnerae* T7901 (YP_003074739) |
| MS48 | 86 | 81 | 2 | *T. turnerae* T7901 (YP_003074739) |
| MS52 | 86 | 77 | 4 | *T. turnerae* T7901 (YP_003074739) |
| MS56 | 86 | 71 | 3 | *T. turnerae* T7901 (YP_003074739) |
| MS79 | 86 | 82 | 3 | *T. turnerae* T7901 (YP_003074739) |
| MS12 | 92 | 55 | 5 | *Thermoanaerobacter italicus* Ab9 (YP_003476082) |
| MS22 | 85 | 60 | 2 | *Thermobaculum terrenum* ATCC BAA-798 (YP_003323207) |
| MS57 | 85 | 62 | 1 | *T. terrenum* ATCC BAA-798 (YP_003323207) |
| MS29 | 84 | 60 | 5 | *Thermobifida alba* (CAB02654) |
| MS27 | 86 | 83 | 5 | *Verrucomicrobiae bacterium* DG1235 (ZP_05056496) |
| MS44 | 86 | 82 | 2 | *V. bacterium* DG1235 (ZP_05056496) |
| MS70 | 86 | 84 | 5 | *V. bacterium* DG1235 (ZP_05056496) |
| MS97 | 86 | 81 | 3 | *V. bacterium* DG1235 (ZP_05056496) |
| MS99 | 86 | 88 | 3 | *V. bacterium* DG1235 (ZP_05056496) |
| Total 48 |  |  | 190 |  |

*^a^* Sequence name was selected to represent each OTU.
