# Supplementary material for: Phylogenetic Diversity and Environment-Specific Distributions of Glycosyl Hydrolase Family 10 Xylanases in Geographically Distant Soils
Source: PLoS One. 2012 Aug 17;7(8):e43480. doi: 10.1371/journal.pone.0043480 (PMC3422244; doi:10.1371/journal.pone.0043480)
Supplement: Table S3 — The GH 10 xylanase gene fragments detected in the pond sediment (PS) and their closest relative based on amino acid sequence identity and similarity. (DOC) [file pone.0043480.s005.doc]

**Supplementary Table S3.** The GH 10 xylanase gene fragments detected in the pond sediment and their closest relatives based on amino acid sequence identity and similarity.

| OTU *^a^* | Protein size (amino acids) | Identity (%) | Amount of sequences | Closest relative (accession No.) |
| --- | --- | --- | --- | --- |
| PS6 | 85 | 88 | 1 | *Amycolatopsis mediterranei* U32 (YP_003766184) |
| PS121 | 98 | 40 | 2 | *Aspergillus aculeatus* (BAA25847) |
| PS36 | 84 | 64 | 3 | *Bacteroides cellulosilyticus* DSM 14838 (ZP_03679080) |
| PS96 | 93 | 66 | 3 | *B. cellulosilyticus* DSM 14838 (ZP_03676788) |
| PS128 | 86 | 65 | 3 | *B. cellulosilyticus* DSM 14838 (ZP_03678239) |
| PS32 | 93 | 61 | 2 | *Bacteroides eggerthii* DSM 20697 (ZP_03459580) |
| PS63 | 93 | 65 | 8 | *B. eggerthii* DSM 20697 (ZP_03459580) |
| PS5 | 88 | 73 | 4 | *Bacteroides intestinalis* DSM 17393 (ZP_03013017) |
| PS17 | 88 | 66 | 2 | *B. intestinalis* DSM 17393 (ZP_03013017) |
| PS45 | 88 | 77 | 2 | *B. intestinalis* DSM 17393 (ZP_03013017) |
| PS56 | 84 | 61 | 1 | *Bacteroides ovatus* ATCC 8483 (ZP_02067380) |
| PS68 | 84 | 58 | 2 | *B. ovatus* ATCC 8483 (ZP_02067380) |
| PS16 | 84 | 61 | 3 | *Bacteroides* sp. D4 (ZP_04557128) |
| PS90 | 84 | 59 | 2 | *Bacteroides xylanisolvens* XB1A (CBH32823) |
| PS137 | 89 | 42 | 2 | *Caldicellulosiruptor kronotskyensis* 2002 (YP_004025163) |
| PS33 | 80 | 42 | 2 | *Clavibacter michiganensis* (YP_001708875) |
| PS108 | 84 | 37 | 3 | *Clostridium cellulolyticum* H10 (YP_002504521) |
| PS13 | 84 | 58 | 2 | *Flavobacteriaceae bacterium* 3519-10 (YP_003096238) |
| PS26 | 84 | 58 | 2 | *F. bacterium* 3519-10 (YP_003096238) |
| PS154 | 86 | 46 | 3 | *Fusarium oxysporum* (BAB88658) |
| PS34 | 84 | 65 | 2 | *Glaciecola mesophila* (ACN76857) |
| PS2 | 82 | 37 | 3 | *Kitasatospora setae* KM-6054 (BAJ26973) |
| PS47 | 95 | 54 | 2 | *Micromonospora aurantiaca* ATCC 27029 (YP_003836747) |
| PS27 | 85 | 85 | 3 | *Micromonospora* sp. ATCC 39149 (ZP_04607260) |
| PS31 | 85 | 56 | 5 | *Mucilaginibacter paludis* DSM 18603 (ZP_07748005) |
| PS75 | 85 | 59 | 2 | *M. paludis* DSM 18603 (ZP_07748005) |
| PS97 | 81 | 54 | 10 | *Opitutaceae bacterium* TAV2 (ZP_03726438) |
| PS10 | 96 | 42 | 2 | *Opitutus terrae* PB90-1 (YP_001820539) |
| PS74 | 94 | 80 | 2 | *Paludibacter propionicigenes* WB4 (YP_004042750) |
| PS84 | 94 | 84 | 2 | *P. propionicigenes* WB4 (YP_004042750) |
| PS88 | 94 | 83 | 2 | *P. propionicigenes* WB4 (YP_004042750) |
| PS125 | 87 | 76 | 3 | *P. propionicigenes* WB4 (YP_004043427) |
| PS159 | 84 | 68 | 30 | *Prevotella bergensis* DSM 17361 (ZP_06006687) |
| PS148 | 84 | 76 | 2 | *Prevotella copri* DSM 18205 (ZP_06252071) |
| PS44 | 84 | 74 | 2 | *P. copri* DSM 18205 (ZP_06252071) |
| PS18 | 106 | 70 | 3 | *Prevotella ruminicola* (AAB81559) |
| PS15 | 97 | 66 | 4 | *P. ruminicola* 23 (YP_003575973) |
| PS21 | 97 | 66 | 3 | *P. ruminicola* 23 (YP_003575973) |
| PS38 | 97 | 68 | 5 | *P. ruminicola* 23 (YP_003575973) |
| PS61 | 92 | 73 | 3 | *P. ruminicola* 23 (YP_003575973) |
| PS65 | 92 | 73 | 2 | *P. ruminicola* 23 (YP_003575973) |
| PS66 | 97 | 74 | 2 | *P. ruminicola* 23 (YP_003575973) |
| PS78 | 97 | 66 | 3 | *P. ruminicola* 23 (YP_003575973) |
| PS92 | 92 | 80 | 6 | *P. ruminicola* 23 (YP_003575973) |
| PS104 | 92 | 76 | 1 | *P. ruminicola* 23 (YP_003575973) |
| PS106 | 97 | 67 | 5 | *P. ruminicola* 23 (YP_003575973) |
| PS111 | 92 | 70 | 9 | *P. ruminicola* 23 (YP_003575973) |
| PS149 | 92 | 73 | 2 | *P. ruminicola* 23 (YP_003575973) |
| PS151 | 92 | 71 | 3 | *P. ruminicola* 23 (YP_003575973) |
| PS155 | 93 | 75 | 3 | *P. ruminicola* 23 (YP_003575973) |
| PS158 | 92 | 73 | 3 | *P. ruminicola* 23 (YP_003575973) |
| PS138 | 97 | 72 | 2 | *P. ruminicola* 23 (YP_003575973) |
| PS126 | 83 | 49 | 2 | *Pseudomonas* sp. PE2 (BAC24105) |
| PS55 | 84 | 68 | 2 | *Solibacter usitatus* Ellin6076 (YP_823955) |
| PS89 | 84 | 69 | 3 | *S. usitatus* Ellin6076 (YP_823955) |
| PS131 | 86 | 53 | 2 | *S. usitatus* Ellin6076 (YP_825640) |
| PS134 | 84 | 58 | 2 | *S. usitatus* Ellin6076 (YP_823955) |
| PS37 | 86 | 78 | 2 | *Teredinibacter turnerae* T7901 (YP_003074739) |
| PS122 | 86 | 60 | 2 | *Thermobacillus xylanilyticus* (CAA76420) |
| PS136 | 85 | 57 | 2 | *Thermobaculum terrenum* ATCC BAA-798 (YP_003323207) |
| PS87 | 91 | 52 | 3 | *Thermotoga* sp. (AAA90913) |
| PS9 | 86 | 80 | 2 | *Verrucomicrobiae bacterium* DG1235 (ZP_05056496) |
| PS28 | 86 | 88 | 3 | *V. bacterium* DG1235 (ZP_05056496) |
| PS53 | 85 | 60 | 2 | *Zunongwangia profunda* SM-A87 (YP_003585554) |
| Total 64 |  |  | 210 |  |

*^a^* Sequence name was selected to represent each OTU.
