# Supplementary material for: Phylogenetic Diversity and Environment-Specific Distributions of Glycosyl Hydrolase Family 10 Xylanases in Geographically Distant Soils
Source: PLoS One. 2012 Aug 17;7(8):e43480. doi: 10.1371/journal.pone.0043480 (PMC3422244; doi:10.1371/journal.pone.0043480)
Supplement: Table S4 — The GH 10 xylanase gene fragments detected in the snow lotus soil (SS) and their closest relative based on amino acid sequence identity and similarity. (DOC) [file pone.0043480.s006.doc]

**Supplementary Table S4.** The GH 10 xylanase gene fragments detected in the snow lotus soil and their closest relatives based on amino acid sequence identity and similarity.

| OTU *^a^* | Protein size (amino acids) | Identity (%) | Amount of sequences | Closest relative (accession No.) |
| --- | --- | --- | --- | --- |
| SS6 | 97 | 41 | 2 | *Aspergillus fumigatus* Af293 (XP_754103) |
| SS8 | 97 | 36 | 6 | *A. fumigatus* Af293 (XP_754103) |
| SS22 | 97 | 39 | 2 | *A. fumigatus* Af293 (XP_754103) |
| SS104 | 97 | 42 | 2 | *A. fumigatus* Af293 (XP_754103) |
| SS28 | 88 | 75 | 4 | *Bacteroides cellulosilyticus* DSM 14838 (ZP_03678239) |
| SS116 | 88 | 75 | 2 | *B. cellulosilyticus* DSM 14838 (ZP_03678239) |
| SS147 | 88 | 73 | 2 | *B. cellulosilyticus* DSM 14838 (ZP_03678239) |
| SS149 | 88 | 45 | 1 | *B. cellulosilyticus* DSM 14838 (ZP_03678239) |
| SS177 | 94 | 65 | 1 | *Bacteroides eggerthii* DSM 20697 (ZP_03459580) |
| SS190 | 93 | 66 | 2 | *B. eggerthii* DSM 20697 (ZP_03459580) |
| SS9 | 88 | 66 | 6 | *Bacteroides intestinalis* DSM 17393 (ZP_03013017) |
| SS108 | 88 | 72 | 1 | *B. intestinalis* DSM 17393 (ZP_03013017) |
| SS210 | 88 | 74 | 2 | *B. intestinalis* DSM 17393 (ZP_03013017) |
| SS4 | 85 | 55 | 2 | *Clostridium papyrosolvens* DSM 2782 (ZP_05496079) |
| SS60 | 86 | 59 | 2 | *Clostridium thermocellum* ATCC 27405 (YP_001038374) |
| SS92 | 84 | 97 | 6 | *Flavobacterium* sp. LW53 (ACN87363) |
| SS138 | 86 | 55 | 1 | *Nectria haematococca* (XP_003040035) |
| SS139 | 83 | 52 | 2 | *O. bacterium* TAV2 (ZP_03726438) |
| SS41 | 83 | 53 | 2 | *Opitutaceae bacterium* TAV2 (ZP_03726438) |
| SS88 | 83 | 47 | 4 | *Opitutus terrae* PB90-1 (YP_001820089) |
| SS129 | 84 | 70 | 1 | *Prevotella bergensis* DSM 17361 (ZP_06006687) |
| SS201 | 84 | 70 | 1 | *Prevotella buccae* ATCC 33574 (ZP_07881708) |
| SS119 | 84 | 71 | 12 | *Prevotella copri* DSM 18205 (ZP_06252071) |
| SS156 | 84 | 74 | 1 | *P. copri* DSM 18205 (ZP_06252071) |
| SS2 | 92 | 77 | 14 | *Prevotella ruminicola* 23 (YP_003575973) |
| SS12 | 97 | 66 | 3 | *P. ruminicola* 23 (YP_003575973) |
| SS20 | 88 | 68 | 3 | *P. ruminicola* 23 (YP_003575973) |
| SS57 | 92 | 78 | 1 | *P. ruminicola* 23 (YP_003575973) |
| SS95 | 97 | 73 | 1 | *P. ruminicola* 23 (YP_003575973) |
| SS97 | 93 | 78 | 2 | *P. ruminicola* 23 (YP_003575973) |
| SS121 | 92 | 78 | 1 | *P. ruminicola* 23 (YP_003575973) |
| SS136 | 97 | 66 | 1 | *P. ruminicola* 23 (YP_003575973) |
| SS148 | 92 | 73 | 9 | *P. ruminicola* 23 (YP_003575973) |
| SS142 | 92 | 76 | 5 | *P. ruminicola* 23 (YP_003575973) |
| SS163 | 92 | 71 | 11 | *P. ruminicola* 23 (YP_003575973) |
| SS184 | 92 | 76 | 3 | *P. ruminicola* 23 (YP_003575973) |
| SS207 | 97 | 63 | 2 | *P. ruminicola* 23 (YP_003575973) |
| SS208 | 97 | 57 | 1 | *P. ruminicola* 23 (YP_003575973) |
| SS183 | 92 | 78 | 1 | *Sorangium cellulosum '*So ce 56' (YP_001617342) |
| SS187 | 93 | 73 | 2 | *S. cellulosum '*So ce 56' (YP_001617342) |
| SS93 | 92 | 55 | 4 | *Spirochaeta thermophila* DSM 6578 (EFU19827) |
| SS159 | 84 | 60 | 2 | *S. thermophila* DSM 6578 (EFU19827) |
| SS72 | 85 | 68 | 2 | *S. thermophila* DSM 6192 (YP_003874977) |
| SS103 | 87 | 56 | 1 | *S. thermophila* DSM 6192 (YP_003874722) |
| SS3 | 84 | 55 | 2 | *Solibacter usitatus* Ellin6076 (YP_823955) |
| SS18 | 84 | 58 | 2 | *S. usitatus* Ellin6076 (YP_823955) |
| SS19 | 84 | 59 | 2 | *S. usitatus* Ellin6076 (YP_823955) |
| SS31 | 84 | 59 | 2 | *S. usitatus* Ellin6076 (YP_823955) |
| SS52 | 84 | 60 | 1 | *S. usitatus* Ellin6076 (YP_823955) |
| SS77 | 84 | 59 | 1 | *S. usitatus* Ellin6076 (YP_823955) |
| SS110 | 84 | 63 | 3 | *S. usitatus* Ellin6076 (YP_823955) |
| SS167 | 85 | 62 | 1 | *Streptomyces pristinaespiralis* ATCC 25486 (ZP_06913485) |
| SS68 | 85 | 76 | 3 | *Streptomyces* sp. C (ZP_05504680) |
| SS15 | 87 | 60 | 6 | *Streptomyces violaceusniger* Tu 4113 (ZP_07608662) |
| SS71 | 86 | 49 | 1 | *Thermoanaerobacterium saccharolyticum* (ADB23440) |
| SS98 | 85 | 57 | 2 | *Thermobaculum terrenum* ATCC BAA-798 (YP_003323207) |
| SS114 | 85 | 42 | 1 | *T. terrenum* ATCC BAA-798 (YP_003323207) |
| SS21 | 87 | 64 | 4 | *Thermobifida alba* (CAB02654) |
| SS32 | 87 | 56 | 5 | *Thermotoga naphthophila* RKU-10 (YP_003346209) |
| SS54 | 86 | 85 | 2 | *Verrucomicrobiae bacterium* DG1235 (ZP_05056496) |
| SS153 | 87 | 73 | 17 | *V. bacterium* DG1235 (ZP_05056496) |
| SS80 | 84 | 68 | 2 | *Zunongwangia profunda* SM-A87 (YP_003583300) |
| Total 63 |  |  | 193 |  |

*^a^* Sequence name was selected to represent each OTU.
