# Supplementary material for: Phylogenetic Diversity and Environment-Specific Distributions of Glycosyl Hydrolase Family 10 Xylanases in Geographically Distant Soils
Source: PLoS One. 2012 Aug 17;7(8):e43480. doi: 10.1371/journal.pone.0043480 (PMC3422244; doi:10.1371/journal.pone.0043480)
Supplement: Table S5 — The GH 10 xylanase gene fragments detected in the farmland soil (FS) and their closest relative based on amino acid sequence identity and similarity. (DOC) [file pone.0043480.s007.doc]

**Table S5.** The GH 10 xylanase gene fragments detected in the farmland soil and their closest relatives based on amino acid sequence identity and similarity.

| OTU *^a^* | Protein size (amino acids) | Identity (%) | Amount of sequences | Closest relative (accession No.) |
| --- | --- | --- | --- | --- |
| FS78 | 85 | 57 | 7 | *Thermotoga naphthophila* RKU-10 (YP_003346209) |
| FS97 | 85 | 62 | 13 | *Ktedonobacter racemifer* DSM 44963 (ZP_06970944) |
| FS102 | 85 | 62 | 3 | *Coprinopsis cinerea* okayama7#130 (XP_001838887) |
| FS46 | 85 | 63 | 2 | *K. racemifer* DSM 44963 (ZP_06970944) |
| FS124 | 85 | 62 | 2 | *Herpetosiphon aurantiacus* ATCC 23779 (YP_001547564) |
| **FS130** | **86** | **59** | **2** | ***Halorhabdus utahensis* DSM 12940 (YP_003130024)** |
| FS134 | 96 | 81 | 2 | *Acidothermus cellulolyticus* 11B (YP_872132) |
| FS43 | 85 | 69 | 3 | *Thermobaculum terrenum* ATCC BAA-798 YP_003323207) |
| FS54 | 91 | 51 | 2 | *T. errenum* ATCC BAA-798 (YP_003323207) |
| FS125 | 87 | 59 | 2 | *Streptomyces violaceusniger* Tu 4113 (ZP_07608662) |
| FS89 | 93 | 69 | 8 | *Sorangium cellulosum* 'So ce 56' (YP_001617342) |
| FS79 | 93 | 74 | 2 | *S. cellulosum* 'So ce 56' (YP_001617342) |
| FS75 | 93 | 62 | 2 | *S. cellulosum* 'So ce 56' (YP_001617342) |
| FS8 | 93 | 66 | 2 | *S. cellulosum* 'So ce 56' (YP_001617342) |
| FS113 | 85 | 55 | 2 | *Spirochaeta thermophila* DSM 6578 (EFU19827) |
| FS121 | 86 | 53 | 2 | *S. thermophila* DSM 6578 (EFU19827) |
| FS30 | 91 | 49 | 2 | *Streptomyces viridochromogenes* (ZP_05530068) |
| FS18 | 84 | 80 | 12 | *Spirosoma linguale* DSM 74 (YP_003386253) |
| FS77 | 84 | 75 | 2 | *S. linguale* DSM 74 (YP_003386253) |
| FS40 | 84 | 78 | 2 | *S. linguale* DSM 74 (YP_003386253) |
| FS58 | 90 | 53 | 2 | *Asticcacaulis excentricus* CB 48 (YP_004086479) |
| FS127 | 86 | 62 | 2 | *Solibacter usitatus* Ellin6076 (YP_825640) |
| FS12 | 84 | 57 | 3 | *S. usitatus* Ellin6076 (YP_825640) |
| FS26 | 84 | 58 | 3 | *S. usitatus* Ellin6076 (YP_823955) |
| FS19 | 84 | 61 | 2 | *S. usitatus* Ellin6076 (YP_823955) |
| FS66 | 84 | 61 | 4 | *S. usitatus* Ellin6076 (YP_823955) |
| FS93 | 86 | 58 | 2 | *S. usitatus* Ellin6076 (YP_823955) |
| FS122 | 84 | 64 | 3 | *Leadbetterella byssophila* DSM 17132 (YP_003998360) |
| FS69 | 84 | 65 | 2 | *Prevotella bergensis* DSM 17361 (ZP_06006687) |
| FS31 | 84 | 65 | 2 | *P. bergensis* DSM 17361 (ZP_06006687) |
| FS85 | 86 | 58 | 6 | *Verrucomicrobiae bacterium* DG1235 (ZP_05059119) |
| FS15 | 86 | 74 | 2 | *V. acterium* DG1235 (ZP_05056496) |
| FS33 | 86 | 79 | 2 | *V. acterium* DG1235 (ZP_05056496) |
| FS47 | 86 | 81 | 5 | *V. acterium* DG1235 (ZP_05056496) |
| FS50 | 86 | 85 | 2 | *V. acterium* DG1235 (ZP_05056496) |
| FS86 | 86 | 78 | 2 | *V. acterium* DG1235 (ZP_05056496) |
| FS38 | 86 | 86 | 3 | *V. acterium* DG1235 (ZP_05056496) |
| FS81 | 86 | 78 | 2 | *V. acterium* DG1235 (ZP_05056496) |
| FS107 | 86 | 75 | 2 | *V. acterium* DG1235 (ZP_05056496) |
| FS55 | 86 | 75 | 3 | *V. acterium* DG1235 (ZP_05056496) |
| FS128 | 85 | 73 | 2 | *V. acterium* DG1235 (ZP_05056496) |
| FS32 | 86 | 77 | 2 | *V. acterium* DG1235 (ZP_05056496) |
| FS112 | 86 | 82 | 2 | *Teredinibacter turnerae* T7901 (YP_003074739) |
| FS98 | 86 | 77 | 2 | *T. urnerae* T7901 (YP_003074739) |
| FS63 | 86 | 74 | 4 | *T. urnerae* T7901 (YP_003074739) |
| FS91 | 86 | 68 | 6 | *Dictyoglomus turgidum* DSM 6724 (YP_002353534) |
| FS24 | 86 | 63 | 2 | *D. urgidum* DSM 6724 (YP_002353534) |
| FS141 | 88 | 69 | 2 | *Bacteroides cellulosilyticus* DSM 14838 (ZP_03678239) |
| FS140 | 88 | 72 | 2 | *Bacteroides intestinalis* DSM 17393 (ZP_03013017) |
| FS138 | 97 | 67 | 3 | *Prevotella ruminicola* 23 (YP_003575973) |
| FS139 | 90 | 78 | 2 | *P. ruminicola* 23 (YP_003575973) |
| FS144 | 92 | 79 | 2 | *P. ruminicola* 23 (YP_003575973) |
| FS143 | 84 | 74 | 3 | *Prevotella copri* DSM 18205 (ZP_06252071) |
| FS23 | 85 | 62 | 3 | *Streptomyces pristinaespiralis* ATCC 25486 (ZP_06913485) |
| FS80 | 89 | 61 | 8 | *Paludibacter propionicigenes* WB4 (YP_004042750) |
| FS115 | 94 | 83 | 4 | *P. ropionicigenes* WB4 (YP_004042750) |
| FS116 | 94 | 82 | 2 | *P. ropionicigenes* WB4 (YP_004042750) |
| FS114 | 94 | 74 | 2 | *P. ropionicigenes* WB4 (YP_004042750) |
| FS51 | 89 | 67 | 2 | *P. ropionicigenes* WB4 (YP_004042750) |
| FS22 | 92 | 62 | 2 | *P. ropionicigenes* WB4 (YP_004042750) |
| FS48 | 97 | 42 | 3 | *Opitutaceae bacterium TAV2* (ZP_03726438) |
| Total 61 |  |  | 188 |  |

*^a^* Sequence name was selected to represent each OTU.
